# Complete genome sequence of the lytic *Pseudomonas fluorescens *phage ϕIBB-PF7A

**DOI:** 10.1186/1743-422X-8-142

**Published:** 2011-03-26

**Authors:** Sanna Sillankorva, Leon D Kluskens, Erika J Lingohr, Andrew M Kropinski, Peter Neubauer, Joana Azeredo

**Affiliations:** 1 IBB-Institute for Biotechnology and Bioengineering, Centre of Biological Engineering, Universidade do Minho, Campus de Gualtar 4710-057, Braga, Portugal; 2 Bioprocess Engineering Laboratory, Department of Process and Environmental Engineering and Biocenter Oulu, P.O. Box 4300, FIN-90014 University of Oulu, Finland; 3 Public Health Agency of Canada, Laboratory for Foodborne Zoonoses, Guelph, Ontario N1G 3W4, Canada; 4 Department of Molecular and Cellular Biology, University of Guelph, Guelph, Ontario N1G 2W1, Canada; 5 Department of Biotechnology, Technische Universität Berlin, Berlin, Germany

## Abstract

**Background:**

Phage ϕIBB-PF7A is a T7-like bacteriophage capable of infecting several *Pseudomonas fluorescens *dairy isolates and is extremely efficient in lysing this bacterium even when growing in biofilms attached to surfaces. This work describes the complete genome sequence of this phage.

**Results:**

The genome consists of a linear double-stranded DNA of 40,973 bp, with 985 bp long direct terminal repeats and a GC content of approximately 56%. There are 52 open reading frames which occupy 94.6% of the genome ranging from 137 to 3995 nucleotides. Twenty eight (46.7%) of the proteins encoded by this virus exhibit sequence similarity to coliphage T7 proteins while 34 (81.0%) are similar to proteins of *Pseudomonas *phage gh-1.

**Conclusions:**

That this phage is closely related to *Pseudomonas putida *phage gh-1 and coliphage T7 places it in the "T7-like viruses" genus of the subfamily *Autographivirinae *within the family *Podoviridae*. Compared to the genome of gh-1, the sequence of ϕIBB-PF7A is longer and contains more genes with unassigned function and lacks a few potentially essential and non-essential T7 genes, such as gene1.1, 3.8, and 7.

## Background

Formerly, the phylogenetic and taxonomic classification of bacteriophages (phages) was based on similarities in phage morphology, host range [1], ability to recombine, and also on similar cross-hybridization patterns of their DNAs [2-4]. Today, phage genome sequencing is commonly performed for classification and characterization purposes and is based on the arrangement of conserved genes and the nucleotide and protein sequence identity [5,6]. A specific group of phages has a number of conserved proteins but also a relatively high number of proteins for which no match occurs in the current databases. This reveals the great genetic diversity existent within phages. Of the total global estimated phage population (> 10^31^), only a small number have been completely sequenced.

In the last few years, a large number of T7-like phages have been sequenced most of which have been gathered in a new viral subfamily within the family *Podoviridae*. The *Autographivirinae *was formerly referred to as the T7 supergroup of phages [7]. All these phages, most of which have members of the *Enterobacteriaceae *as their host, present a highly conserved genome organization and mainly differ at sequence level through the presence or absence of nonessential genes [8]. Phages presenting more similarities to each other are grouped in genera within the *Autographivirinae*, namely the "T7-like viruses", the "SP6-like viruses" and the "phiKMV-like viruses". The genome of coliphage T7 has 56 genes encoding potential proteins [8], of which 35 have known function or functions. In addition, there are 25 nonessential proteins, of which only 12 are conserved across the genus "T7-like viruses" [8].

In this manuscript we report on the full genomic characterization of *Pseudomonas fluorescens *phage ϕIBB-PF7A, a phage capable of infecting several dairy *P. fluorescens *isolates including isolates belonging to different ribotypes [9]. On the basis of TEM micrographs this virus resembled T7-like phages with a head diameter of about 63 nm and a noncontractile tail size approximately 13 × 8 nm. In terms of growth cycle, phage ϕIBB-PF7A has a latent period of 15 minutes, an eclipse period of 10 minutes and a burst size of 153 plaque forming units per infected cell. Furthermore, this phage has been the focus of several recent studies lysing efficiently its host present in single [10] and dual species biofilms [11]. Moreover, ϕIBB-PF7A is the first reported phage capable of efficiently infecting elongated *P. fluorescens *cells - which are up to 10 times longer than normal sized cells - and killing planktonic stationary (3 days old) phase cells [9]. For these reasons, a more detailed characterization was desired in order to further increase our knowledge about this particular lytic phage.

## Results and Discussion

This work focuses on the determination of the complete genome sequence of the *P. fluorescens *phage ϕIBB-PF7A. This virus is the first T7-like bacteriophage for *P. fluorescens *for which the genome has been determined.

### ϕIBB-PF7A nucleotide sequence and general sequence properties

The DNA sequence of phage ϕIBB-PF7A was determined and it consists of linear double-stranded DNA of 40,973 bp. The size of this phage correlates well with other T7-like phage members, where the smallest and largest phages reported so far are the *Pseudomonas putida *phage gh-1 (37.4 kb) and the *Vibrio parahaemolyticus *phage VpV262 (45.9 kb), respectively [12]. The redundant terminal repeats (DTR) of ϕIBB-PF7A are unusually long (985 bp) for member of the T7 group (T7 has DTRs of only 160 bp [13]) and, up till now, the genome of *P. aeruginosa *phage LKD16 had the longest DTRs (428 bp) [14]. Phage ϕIBB-PF7A has an overall genomic guanine plus cytosine (GC) content of 56.3%, well within the range of GC contents observed in other T7-like phages (46.2 - 62.3%) [15].

The genome of ϕIBB-PF7A was scanned for open reading frames (ORFs) of 100 bp or longer using computational software complemented with visual inspection of the genome sequence. The search resulted in 52 predicted genes with lengths ranging from 137 to 3995 nt. The initiation codon of 92% of the protein-coding genes is ATG and only four other ORFs, including RNA polymerase (gene1) and the packaging protein B (gene19), start with GTG. The temporal and functional distributions of genes are tightly organized and packed close to each other so that they occupy 94.6% of the entire nucleotide sequence.

### Comparative genomics of phage ϕIBB-PF7A

All predicted protein-coding genes were screening using BLASTP against the non-redundant protein database at NCBI. From the 52 ORFs of ϕIBB-PF7A: i) 25 (48.1%) have assigned function; ii) 12 (23.1%) are similar to proteins of unknown function in the nonredundant databases and (iii) 15 (28.8%) code for hypothetical proteins unique to this bacteriophage. While the great majority (34) of the homologs is to proteins of *Pseudomonas *phage gh-1, examples of primary sequence similarity to *Pseudomonas *phages phi-2 and LKA1, coliphage T3, *Yersinia *phage φYeO3-12, *Vibrio *phage ICP3_2008_A and *Kluyvera *phage Kvp1 exist. With the exceptions of *Pseudomonas *phage LKA1 which is a member of the "phiKMV-like viruses," all of these phages are members of the "T7-like viruses" genus. Since 28 gene products were related to T7 phage proteins, the T7 gene nomenclature was adopted for these genes (Additional file 1, Table S1). No function can be speculated about the hypothetical proteins of phage ϕIBB-PF7A without further study. Based upon overall protein homology determined using CoreGenes [16,17] this virus shares 28 (47%), 11 (21%) and 10 (20%), similar proteins with phages T7, SP6 and φKMV, respectively. These results indicate that it is a member of the *Autographivirinae*, specifically a member of the "T7-like viruses" genus [7].

**Figure 1:**
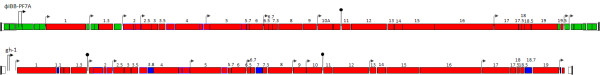
**Genomic map of *P. fluorescens *phage ϕIBB-PF7A and *P. putida *phage gh-1**. Filled green boxes are hypothetical proteins unique of ϕIBB-PF7A, red filled boxes with black and blue outlines indicate genes of ϕIBB-PF7A which show homology to conserved essential genes of and to nonessential conserved genes of gh-1, blue filled boxes are genes present only in phage gh-1. Putative transcription promoters are indicated by lines with arrowhead: black - early host RNA polymerase promoters; dark grey - phage RNA promoters. Lines with filled black spheres indicate ρ-independent terminators. Unfilled dark grey outlined boxes indicate direct terminal repeats and dark lines at left and right indicate the ends of the genome.

At the protein level ϕIBB-PF7A showed the greatest sequence identity with proteins of phage gh-1, and therefore the gene clustering of the two phages was compared (Figure 1). Unlike *P. putida *phage gh-1, which presents no putative genes before gene 1, in ϕIBB-PF7A we observed six unique hypothetical proteins of which two (orf1 and orf2) are part of the 985 bp long DTR. Furthermore, these first six ORFs do not display sequence similarity to any of the T7 genes between gene0.3 and gene0.7. Compared to gh-1, phage ϕIBB-PF7A also lacks gene 1.1, a gene with unknown function.

Despite the different host ranges of *P. fluorescens *phage ϕIBB-PF7A and *P. putida *phage gh-1, there are 34 phage proteins which show significant sequence identity (Figure 1). As pointed out above, phage gh-1 belongs to the same viral genus as ϕIBB-PF7A and its sequence is linear double-stranded DNA of 37,359 bp with 216 bp direct terminal repeats, and has 43 predicted putative genes.

A Mauve alignment [16,18] of phage ϕIBB-PF7A DNA against the genomes of gh-1, T3 and T7 show the regions of DNA sequence similarity (colored areas) interspersed with regions (white) where no sequence similarity exists (Additional file 2; Figure S1).

### DNA penetration

The genes essential of forming the channel which allows the phage DNA injection into the hosts' cytoplasm are all present in phage ϕIBB-PF7A, namely gene14, gene15 and gene16.

### ϕIBB-PF7A gene arrangement

#### A. Early gene expression

The early genes of this type of virus are transcribed by the host RNA polymerase. The transcribed genes include genes which function to overcome host restriction and to convert the metabolism of the host cell to the production of phage proteins, e.g. synthesis of the phage promoter-specific RNA polymerase (RNAP; gene1). Two host-type promoters were discovered (Additional file 3; Table S2). Interestingly, the early region of this phage contains six ORFs, two of which are in the LTR, which show no sequence similarity at the DNA or protein level to the early genes of any other member of the *Autographivirinae*. Indeed, this is the first report for the existence of putative ORFs within the terminal repeats of a member of this subfamily, though they are found in phages such as T5 and SPO1. ϕIBB-PF7A does not encode the T7-like Gp0.3 (ocr), 0.6 or 0.7 homologs.

#### B. Middle gene expression

The class II genes are transcribed by the phage RNAP and are involved in DNA replication. Both phages ϕIBB-PF7A and gh-1 lack several class II genes present in T7, namely: gene1.4, gene1.5, gene1.6, gene1.7, gene1.8, gene3.2, gene3.7, gene4.3, and gene5.5. Most of these class II genes are nonessential and only gene1.7 is considered an essential gene and reportedly is beneficial for phage growth [8]. Besides the genes not present in both ϕIBB-PF7A and gh-1, ϕIBB-PF7A is also missing the nonessential homing endonuclease (gene3.8).

#### C. Late gene expression

The late genes or class III genes are transcribed by the phage RNAP and are mostly involved in morphogenesis and host lysis. The majority of putative genes found in T7-like phages are also present in ϕIBB-PF7A (Additional file 1, Table S1). Phage ϕIBB-PF7A is only missing gene7, a nonessential protein which is usually involved in host range [8]. Compared to gh-1, phage ϕIBB-PF7A has more ORFs situated just before the right DTR (Figure 1). We have identified two ORFs which display sequence similarity to the major capsid proteins of phages belonging to the "T7-like viruses." The upstream gene displays homology to the capsid gene of *Yersinia *phage φYeO3-12 while the downstream gene is similar to the analogous protein in *P. putida *phage gh-1. That these two genes might be coexpressed as a single fusion protein is suggested by the presence of a slippery site (GTTATCGAAAAGGCGTAA) which is translated as VIEKA(stop); but could be extended, via -1 frameshifting, into VIEKgvsvpdp...(stop). While a diagnostic pseudoknot was discovered immediately downstream of the potential slippery site [19-22] there is no proteomic evidence for frameshifting in this bacteriophage [9]. The structural proteome of this phage is therefore made up of gene10A (capsid), the connector gene8, and a conspicuous core made by the proteins gene14, gene15, and gene16 [23].

Phage ϕIBB-PF7A contains gene16, the internal virion protein D, which contains peptidoglycan hydrolase motif. This activity is essential for enlarging a hole across the cell wall of cells when hosts are infected at low temperatures [8]. Possibly, due to the presence of this protein phage ϕIBB-PF7A showed a high efficiency towards mature biofilms [10].

### Lysis genes

Endolysins are muralytic enzymes produced by dsDNA phages which hydrolyze the peptidoglycan layer of bacterial cell walls. As in T7 phages, gene3.5 of ϕIBB-PF7A is proposed to be the endolysin-like protein which possesses N-acetylmuramoyl-L-alanine amidase activity, but is apparently not essential for lysis (I. Molineux, personal communication). The access of endolysins to the cell wall occurs through the presence of a secondary lysis factor known as holin. The small membrane protein derived from gene17.5 of ϕIBB-PF7A is proposed as holin and it showed highest sequence similarity to *Morganella *phage MmP1 lysis protein. Phage ϕIBB-PF7A has also one more lysis gene, the λ Rz-like lysis protein (gene18.5) which presents a 34% sequence identity to the homologous protein in phage gh-1. The proteins of phage ϕIBB-PF7A was scanned for transmembrane helices using TMHMM. A total of six ORFS contain transmembrane helices (TMD, orf2, gene17.5, gene18.5, gene19, and gene19.5, and orf52), with only one (gene17.5) presenting two TMD.

### Putative regulatory elements

No tRNA genes were predicted in the ϕIBB-PF7A genome using tRNAscan-SE. Several potential putative promoters were identified in the DNA sequence of ϕIBB-PF7A, using PHIRE and BPROM (Additional file 3, Table S2). Using strict criteria for the assignment of host promoter sites, i.e. that four out of the six bp in the -10 and -35 regions be conserved, two host-dependent promoters were discovered. These are both located in orf4. We have been unable to definitively assign promoters to earlier ORFs.

**Figure 2:**
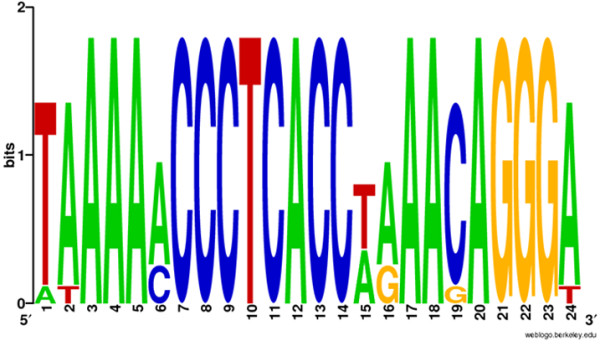
**WebLogo of the phage specific promoter sequences of ϕIBB-PF7A**. WebLogo was created at http://weblogo.berkeley.edu/logo.cgi.

In all T7 group phages, the phage RNA polymerase (RNAP) is responsible for the recognition of phage-specific promoters. In phage ϕIBB-PF7A, we identified 12 phage RNAP specific promoters (Additional file 3, Table S2) which are named according to the downstream gene. Eleven promoters were identified which show remarkable sequence conservation with a consensus of taAAAtmCCCTCACCwrAAcAGGGa (Figure 2). Interestingly the consensus sequence is 1 bp longer than that for the other members of this genus.

The promoter sequences all lie in intergenic regions and show the greatest sequence similarity to those of coliphage T3 (Additional file 3, Table S2). The specificity of the RNAP is determined by the positions -10 and -11 and it is the position -2 that establishes the promoter strength [24]. The highest relative utilization of the promoter is observed when there is a T at the -2 position. Phage ϕIBB-PF7A may have lower promoter utilization since there is not a T positioned at -2. Nonetheless, the promoter positions -10 and -11 are identical to the consensus sequence of T3 and SP6 (C s, respectively). In phage ϕIBB-PF7A, seven of the promoters were found to be present in identical places as in phage T7: ϕ1, ϕ2.5, ϕ6.5, ϕ9, ϕ10A, ϕ17and ϕ19.5, respectively. The promoter sequences which were found in analogous genomic positions to the ones in T7 suggest that the regulation of phage mRNA synthesis is well conserved within the group.

FindTerm, RibEx and TransTerm programs were used to search for transcriptional terminators. T7 and gh-1 phages have three terminators identified while phage ϕIBB-PF7A has only one rho-independent terminator downstream of gene10 (22995 to 23040) which suggest the presence of the Tø2 terminators (named T_Late1 _in phage gh-1). Downstream of gene1.3 is a sequence (gccctgtaccttgcagggc) which exhibits a ΔG of -11.60 kcal/mol. While this sequence is identically placed to the rho-independent terminator of most T7-like phages it lacks the classical polyT tail.

Summarizing, the genome sequencing of ϕIBB-PF7A showed similarity to "T7-like viruses" of the subfamily *Autographivirinae *and allowed the recognition of a new subgroup consisting, so far, of only phages ϕIBB-PF7A and gh-1.

## Methods

### Bacteria and bacteriophage ϕIBB-PF7A

The bacterium used (*P. fluorescens *PF7) was isolated from a dairy industry (Estação Experimental, Paços de Ferreira, Portugal), identified using the API 20NE panel (BioMérieux S.A., France), and grown at 30°C in Tryptic Soy Broth (TSB, Fluka) or TSB agar plates for bacteriophage propagation. Bacteriophage ϕIBB-PF7A was isolated from raw sewage and has been previously characterized according to morphology, growth cycle and other parameters [9].

### Bacteriophage ϕIBB-PF7A propagation

For phage amplification, the plate lysis and elution method described by Sambrook & Russell [25] was used with some modifications. Briefly, 1 ml of phage (1 × 10^3 ^PFU ml^-1^) and 1 ml of overnight grown host were mixed with 30 ml of TSB soft agar (TSB + 0.6% agar) and added to 250 ml T-flasks with a thin layer of TSA media. The soft-agar layer was allowed to solidify and the flasks were incubated at 30°C for 7 h. The phage particles were subsequently eluted with SM buffer and concentrated with PEG 8000, and then extracted with chloroform. Samples in SM buffer were stored at 4°C until further use.

### DNA isolation

Phage DNA was isolated using a Wizard Lambda Preps DNA purification system (Promega, Madison, WI.) according to the manufacturer's instructions.

### Phage DNA sequencing

Phage ϕIBB-PF7A DNA was sheared with a nebulizer. Following 1% agarose gel electrophoresis overnight electrophoresis at 30 V, fractions with sizes between 1 and 4 kb were cut from the gels and the DNA purified. The ends were repaired, the fragments were ligated overnight, at 16°C, into pZero cloning vector (Zero background/Kan cloning kit, Invitrogen, Carlsbad, CA) previously digested with EcoRV, and transformed into chemically competent One Shot^® ^TOP10 cells (Invitrogen). The cells were recovered in SOC medium, incubated for 1 h at 37°C and 225 rpm (Titramax plate shaker, Heidolph Elektro GmbH & Co. KG, Kelheim, Germany) and plated onto LB-Kan plates containing X-Gal. Selected clones were picked with a Genetix QPix2 robotic colony picker (Genetix Corp., Boston, MA) and grown overnight in LB medium at 37°C and 225 rpm. The cultures were centrifuged and plasmid DNA was extracted. For sequencing, Big Dye chemistry was used and the sequencing was carried out in ABI 3700 DNA Sequencer (Applied Biosystems). Sequence reads were assembled using GAP4 (Staden package software) and the sequences were assembled into contigs [26].

Several frameshift errors were corrected, through a primer-walking technique with 20-mer oligonucleotide primers and pure ϕIBB-PF7A DNA with the amplicons being sequenced.

### ORF prediction and annotation

Prediction of open reading frames (ORFs) was performed using GeneMark.hmm, OrfFinder and Kodon (Applied Maths, Austin, TX). For tRNA gene identification, the tRNAscan-SE program was used [27].

Translated ORFs were compared with known protein sequences using BLASTP, against the non-redundant protein GenBank database. To tease out function conserved hypothetical proteins were also analyzed by HHPred at http://toolkit.tuebingen.mpg.de/hhpred.

Molecular masses and isoelectric points of phage ϕIBB-PF7A translated putative proteins were determined using the ExPASy Compute pI/Mw tool http://au.expasy.org/tools/pi_tool.html. Promoter predictions were made using promoter predictor http://www.fruitfly.org/seq_tools/promoter.html, PHIRE 1.0 [28] and BPROM http://linux1.softberry.com/berry.phtml?topic=bprom&group=programs&subgroup=gfindb and the search for transmembrane helices was done by using TMHMM http://www.cbs.dtu.dk/services/TMHMM-2.0/. Terminators were predicted using FindTerm http://linux1.softberry.com/berry.phtml?topic=findterm&group=programs&subgroup=gfindb, RibEx http://132.248.32.45:8080/cgi-bin/ribex.cgi and TransTerm http://nbc11.biologie.uni-kl.de/framed/left/menu/auto/right/transterm/.

### Nucleotide sequence accession number

The GenBank accession number of the complete genomic sequence of phage ϕIBB-PF7A is GU583987.

## Competing interests

The authors declare that they have no competing interests.

## Authors' contributions

SS performed the experiments, annotated the genome and prepared the manuscript, LDK and AMK contributed to the genome annotation; AMK also contributed to manuscript preparation; EJL corrected the errors in the genome sequence; JA and PN supervised the work. The final manuscript was read and accepted by all co-authors.

## Supplementary Material

Additional file 1 **Table S1 Features of phage φIBB-PF7A open reading frames and their homology to other phage proteins**. Supplementary table Click here for file

Additional file 2 **Figure S1 Figure showing progressive Mauve alignment of the genomes of *P. fluorescens *phage ϕIBB-PF7A, *P. putida *phage gh-1, coliphages T3 and T7**. Supplementary figure. Click here for file

Additional file 3 **Table S2 Predicted promoter sequences using PHIRE and BPROM**. Supplementary table Click here for file
