# Supplementary figures and images for: Complete genome sequence of the lytic Pseudomonas fluorescens phage ϕIBB-PF7A
Source: Virol J. 2011 Mar 26;8:142. doi: 10.1186/1743-422X-8-142 (PMC3080317; doi:10.1186/1743-422X-8-142)

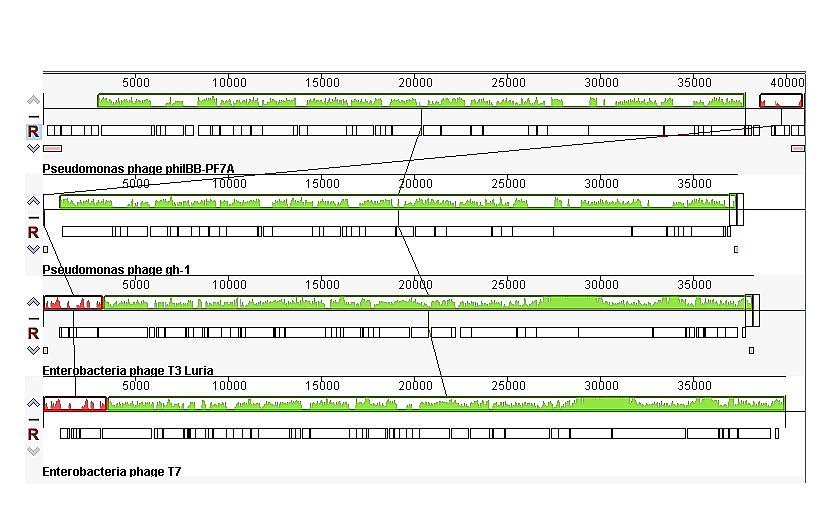

Supplement: Additional file 1 — Table S1 Features of phage φIBB-PF7A open reading frames and their homology to other phage proteins. Supplementary table [file 1743-422X-8-142-S1.TIFF]
