# Supplementary material for: Complete genome sequence of the lytic Pseudomonas fluorescens phage ϕIBB-PF7A
Source: Virol J. 2011 Mar 26;8:142. doi: 10.1186/1743-422X-8-142 (PMC3080317; doi:10.1186/1743-422X-8-142)
Supplement: Additional file 2 — Figure S1 Figure showing progressive Mauve alignment of the genomes of P. fluorescens phage ϕIBB-PF7A, P. putida phage gh-1, coliphages T3 and T7. Supplementary figure. [file 1743-422X-8-142-S2.DOC]

**Supplementary table 1.** Features of phage φIBB-PF7A open reading frames and their homology to other phage proteins.

| **Gene name** | **NCBI locus tag** | **Left end** | **Right end** | **Gene length** | **Protein mass** | **pI** | **No. AA residues** | **Function** | **Homologs & Motifs** | **Expect value** |
| --- | --- | --- | --- | --- | --- | --- | --- | --- | --- | --- |
| orf1 | phiIBB-PF7Ap00 | 252 | 602 | 351 | 12594 | 9.9 | 116 | hypothetical |  |  |
| orf2 | phiIBB-PF7Ap01 | 602 | 928 | 327 | 11631 | 9.1 | 108 | hypothetical | 1 TMD |  |
| orf3 | phiIBB-PF7Ap02 | 996 | 1511 | 516 | 19369 | 9.2 | 171 | hypothetical |  |  |
| orf4 | phiIBB-PF7Ap03 | 1508 | 2215 | 708 | 26375 | 4.7 | 235 | conserved | YP_003345473.1| hypothetical protein SBWP25_0008 [Pseudomonas phage phi-2] | 5E-10 |
| orf5 | phiIBB-PF7Ap04 | 2217 | 2594 | 378 | 13575 | 9.5 | 125 | hypothetical |  |  |
| orf6 | phiIBB-PF7Ap05 | 2591 | 2989 | 399 | 15211 | 9.3 | 132 | hypothetical |  |  |
| **gene1** | phiIBB-PF7Ap06 | 3123 | 5798 | 2676 | 100471 | 6.8 | 891 | RNA polymerase | NP_813747.1| T3/T7-like RNA polymerase [Pseudomonas phage gh-1]; PF00940.12 RNA_pol | 0 |
| orf8 | phiIBB-PF7Ap07 | 5808 | 6002 | 195 | 7420 | 7.9 | 64 | hypothetical |  |  |
| orf9 | phiIBB-PF7Ap08 | 6067 | 6297 | 231 | 8667 | 9.9 | 76 | hypothetical |  |  |
| orf10 | phiIBB-PF7Ap08A | 6294 | 6572 | 279 | 10447 | 8.9 | 92 | hypothetical |  |  |
| **gene1.3** | phiIBB-PF7Ap09 | 6573 | 7556 | 984 | 36826 | 5.3 | 327 | DNA ligase | NP_813751.1| ATP-dependent DNA ligase [Pseudomonas phage gh-1]; PF01068.14 DNA_ligase_A_M | 2E-70 |
| orf12 | phiIBB-PF7Ap10 | 7635 | 8099 | 465 | 17617 | 9.5 | 154 | hypothetical |  |  |
| orf13 | phiIBB-PF7p11 | 8323 | 8988 | 666 | 25612 | 5.9 | 221 | conserved; putative deoxy-nucleoside mono-phosphate kinase (HHPred) | NP_813752.1| hypothetical protein gh-1p06 [Pseudomonas phage gh-1] | 5E-46 |
| **gene2** | phiIBB-PF7Ap11A | 8985 | 9122 | 138 | 4938 | 4.5 | 45 | host RNA-polymerase inhibitor | Psi-BLAST - NP_813754.1| putative host RNA-polymerase inhibitor [Pseudomonas phage gh-1] | 2E-07 |
| orf15 | phiIBB-PF7Ap12 | 9119 | 9460 | 342 | 13170 | 9.8 | 113 | conserved | NP_813755.1| hypothetical protein gh-1p09 [Pseudomonas phage gh-1] | 2E-20 |
| **gene2.5** | phiIBB-PF7Ap13 | 9536 | 10231 | 696 | 25487 | 4.8 | 231 | single-stranded DNA-binding protein | NP_523311.1| single-stranded DNA-binding protein [Enterobacteria phage T3] | 8E-52 |
| **gene3** | phiIBB-PF7Ap14 | 10232 | 10669 | 438 | 16380 | 9.6 | 145 | putative endo-nuclease I | NP_813757.1| endonuclease I [Pseudomonas phage gh-1]; PF05367.4 Phage_endo_I | 6E-41 |
| **gene3.5** | phiIBB-PF7Ap15 | 10682 | 11143 | 462 | 17110 | 8.8 | 153 | N-acetyl-muramoyl-L-alanine amidase | NP_813758.1| lysozyme [Pseudomonas phage gh-1]; PF01510.18 Amidase_2 | 2E-45 |
| orf19 | phiIBB-PF7Ap16 | 11209 | 11772 | 564 | 20913 | 4.8 | 187 | conserved; putative pcnB poly(A) polymerase (HHPred) | NP_813759.1| hypothetical protein gh-1p13 [Pseudomonas phage gh-1] | 4E-22 |
| **gene4** | phiIBB-PF7Ap17 | 11784 | 13475 | 1692 | 62318 | 5.6 | 563 | DNA primase/ helicase | NP_813761.1| putative primase/helicase [Pseudomonas phage gh-1]; PF08273.5 Prim_Zn_Ribbon | 0 |
| orf21 | phiIBB-PF7Ap17A | 13478 | 13696 | 219 | 8018 | 11.0 | 72 | conserved | NP_813762.1| hypothetical protein gh-1p16 [Pseudomonas phage gh-1]; PF04192.5 Utp21 | 8E-04 |
| orf22 | phiIBB-PF7Ap18 | 13755 | 14187 | 433 | 16688 | 8.8 | 147 | conserved | NP_813763.1| hypothetical protein gh-1p17 [Pseudomonas phage gh-1] | 4E-05 |
| **gene5** | phiIBB-PF7Ap19 | 14185 | 16323 | 2139 | 79691 | 6.4 | 712 | DNA polymerase | NP_813764.1| putative DNA polymerase [Pseudomonas phage gh-1]; PF00476.13 DNA_pol_A | 0 |
| orf24 | phiIBB-PF7Ap19A | 16325 | 16639 | 315 | 11454 | 4.8 | 104 | conserved | NP_813765.1| hypothetical protein gh-1p19 [Pseudomonas phage gh-1] | 0.012 |
| **gene5.7** | phiIBB-PF7Ap20 | 16642 | 16851 | 210 | 7703 | 9.6 | 69 | conserved | NP_813766.1| hypothetical protein gh-1p20 [Pseudomonas phage gh-1] | 3E-16 |
| **gene6** | phiIBB-PF7Ap21 | 16848 | 17762 | 915 | 34398 | 4.8 | 304 | exonuclease | NP_813767.1| exonuclease [Pseudomonas phage gh-1] | 2E-86 |
| **gene6.5** | phiIBB-PF7Ap22 | 17870 | 18136 | 267 | 9949 | 4.4 | 88 | conserved | NP_813768.1| hypothetical protein gh-1p22 [Pseudomonas phage gh-1]; PF10911.1 DUF2717 | 1E-08 |
| **gene6.7** | phiIBB-PF7Ap23 | 18123 | 18413 | 291 | 9969 | 9.4 | 96 | conserved | NP_813769.1| hypothetical protein gh-1p23 [Pseudomonas phage gh-1] | 3E-10 |
| **gene7.3** | phiIBB-PF7Ap24 | 18423 | 18743 | 321 | 10800 | 9.8 | 106 | tail assembly protein | NP_813771.1| tail assembly protein [Pseudomonas phage gh-1]; PF11653.1 VirionAssem_T7; PHA00437, tail assembly protein | 0.009 |
| **gene8** | phiIBB-PF7Ap25 | 18756 | 20363 | 1608 | 59108 | 4.7 | 535 | head to tail joining protein | NP_813772.1| head-tail connector protein [Pseudomonas phage gh-1]; PF12236.1 Head-tail_con | 0 |
| **gene9** | phiIBB-PF7Ap26 | 20429 | 21343 | 915 | 32673 | 4.2 | 304 | capsid assembly protein | NP_813773.1| capsid assembly protein [Pseudomonas phage gh-1]; PF05396.4 Phage_T7_Capsid | 3E-66 |
| **gene10A** | phiIBB-PF7Ap27 | 21432 | 22457 | 1026 | 54501 | 5.9 | 519 | major capsid protein | NP_052108.1| minor capsid protein 10B [Yersinia phage phiYeO3-12]; PF02368.11 Big_2 | E-142 |
| **gene10B** | phiIBB-PF7Ap28 | 22499 | 22987 | 489 | 36319 | 6.4 | 341 | minor capsid protein 10B | NP_813774.1| major capsid protein [Pseudomonas phage gh-1] | E-132 |
| **gene11** | phiIBB-PF7Ap29 | 23052 | 23639 | 588 | 22177 | 4.6 | 195 | tail tubular protein A | NP_813775.1| tail tubular protein A [Pseudomonas phage gh-1] | 3E-77 |
| **gene12** | phiIBB-PF7Ap30 | 23650 | 26073 | 2424 | 89421 | 5.7 | 807 | tail tubular protein B | NP_813776.1| tail tubular protein B [Pseudomonas phage gh-1] | 0 |
| **gene13** | phiIBB-PF7Ap31 | 26102 | 26539 | 438 | 16790 | 7.0 | 145 | internal virion protein A | NP_813777.1| internal virion protein A [Pseudomonas phage gh-1]; PF11090.1 DUF2833 | 2E-21 |
| **gene14** | phiIBB-PF7Ap32 | 26551 | 27108 | 558 | 19475 | 6.9 | 185 | internal virion protein B | NP_813778.1| internal virion protein B [Pseudomonas phage gh-1] | 2E-27 |
| **gene15** | phiIBB-PF7Ap33 | 27117 | 29330 | 2214 | 82654 | 5.3 | 737 | internal virion protein C | NP_813779.1| internal virion protein C [Pseudomonas phage gh-1] | E-166 |
| **gene16** | phiIBB-PF7Ap34 | 29334 | 33329 | 3996 | 144778 | 6.5 | 1331 | internal virion protein D | NP_813780.1| internal virion protein D [Pseudomonas phage gh-1]; PF01464.13 SLT | 0 |
| **gene17** | phiIBB-PF7Ap35 | 33392 | 35029 | 1638 | 57005 | 6.1 | 545 | tail fiber protein | NP_813781.1| tail fiber protein [Pseudomonas phage gh-1]; PF03906.7 Phage_T7_tail | 5E-66 |
| **gene17.5** | phiIBB-PF7Ap36 | 35039 | 35242 | 204 | 7596 | 8.1 | 67 | lysin protein (type II holin) | ADX87630.1| putative lysis protein [Vibrio phage ICP3_2008_A]; 2 TMD; PF10746.2 Phage_holin_6 | 9E-06 |
| **gene18** | phiIBB-PF7Ap37 | 35235 | 35492 | 258 | 9746 | 4.6 | 85 | DNA packaging protein A | NP_813784.1| DNA packaging protein, small subunit [Pseudomonas phage gh-1]; PF11123.1 DNA_Packaging_2 | 4E-13 |
| **gene18.5** | phiIBB-PF7Ap38 | 35492 | 35929 | 438 | 16201 | 5.5 | 145 | endo-peptidase | NP_813785.1| phage lambda Rz-like lysis protein [Pseudomonas phage gh-1]; 1 TMD | 6E-12 |
| **gene19** | phiIBB-PF7Ap39 | 35926 | 37692 | 1767 | 66100 | 5.5 | 588 | DNA packaging protein B | NP_813786.1| DNA packaging protein B [Pseudomonas phage gh-1]; 1 TMD; Terminase_6[pfam03237], Terminase-like family | 0 |
| orf45 | phiIBB-PF7Ap39A | 37755 | 37916 | 162 | 6288 | 8.2 | 53 | hypothetical |  |  |
| **gene19.5** | phiIBB-PF7Ap40 | 37913 | 38086 | 174 | 5917 | 9.2 | 57 | conserved | YP_002308428.1| hypothetical protein Kvp1_gp47 [Kluyvera phage Kvp1]; 1 TMD; PHA00406[PHA00406], hypothetical protein | 0.001 |
| orf47 | phiIBB-PF7Ap41 | 38171 | 38488 | 318 | 11582 | 9.9 | 105 | hypothetical |  |  |
| orf48 | phiIBB-PF7Ap42 | 39137 | 39322 | 186 | 6912 | 4.3 | 61 | hypothetical |  |  |
| orf49 | phiIBB-PF7Ap43 | 39348 | 39839 | 492 | 18458 | 5.4 | 163 | conserved | YP_001522848.1| hypothetical protein PPLKA1_gp07 [Pseudomonas phage LKA1] | 2E-16 |
| orf50 | phiIBB-PF7Ap44 | 39836 | 40105 | 270 | 9881 | 6.1 | 89 | hypothetical |  |  |
| orf51 | phiIBB-PF7Ap45 | 40240 | 40590 | 351 | 12594 | 9.9 | 116 | hypothetical |  |  |
| orf52 | phiIBB-PF7Ap46 | 40590 | 40916 | 327 | 11631 | 9.1 | 108 | hypothetical | 1 TDM |  |

BLASTP and Pfam searches made February 25, 2011; TMD = transmembrane domains
