# Supplementary material for: Complete genome sequence of the lytic Pseudomonas fluorescens phage ϕIBB-PF7A
Source: Virol J. 2011 Mar 26;8:142. doi: 10.1186/1743-422X-8-142 (PMC3080317; doi:10.1186/1743-422X-8-142)
Supplement: Additional file 3 — Table S2 Predicted promoter sequences using PHIRE and BPROM. Supplementary table [file 1743-422X-8-142-S3.DOC]

**Table 2 -** Predicted promoter sequences using PHIRE and BPROM.

| **Name** | **Promoter sequence** | **Number of mismatches**† | **Transcription** | |
| --- | --- | --- | --- | --- |
|  |  |  | **Beginning** | **End** |
| Host promoter | TTCACCAGCCGCGAAGTCGCCGCTAAGAT |  | 1020 | 1048 |
| Host promoter | TTGACGATGACCAAGCCGCTCAAAT |  | 1462 | 1486 |
| φ1 | aaaAAACCCTCACCAGAACAGGGA | 2 | 2999 | 3021 |
| φorf9 | taaAACCCCTCACCAAAACAGGGT | 2 | 6002 | 6024 |
| φorf13 | taaAAACCCTCACCAAAACAGGGA | 1 | 8260 | 8282 |
| φ2.5 | taaAAACCCTCACCAGAACAGGGA | 1 | 9461 | 9483 |
| φorf19 | taaAAACCCTCACCAGAACAGGGA | 2 | 11144 | 11166 |
| φorf23 | caaATACCCTCACCTAAACAGCTT | 4 | 13958 | 13980 |
| φ6.5 | taaAACCCCTCACCTAAACAGGGA | 2 | 17796 | 17818 |
| φ9 | taaAAACCCTCACCTAAACAGGGA | 1 | 20364 | 20386 |
| φ10A | taaAAACCCTCACCTAAACAGGGA | 1 | 21357 | 21379 |
| φ17 | taaAACCCCTCACCTAAACAGGGA | 2 | 33330 | 33352 |
| φ19.5 | taaAAACCCTCACCTAAAGAGGGA | 3 | 37732 | 37754 |
| φorf48 | ttaAAACCCTCACCTAAACAGGGA | 3 | 38712 | 38734 |
| Degenerate  Consensus | -10 +1  TaAAAtmCCCTCACCwrAAcAGGGa |  |  |  |
| T3* | AATTAACCCTCACTAAAGGGAGA |  |  |  |
| SP6* | AATTAACCCTCACTAAAGGGAGA |  |  |  |
| T7* | ATTTA GGTGACACTATAGAAGNG |  |  |  |

† Mismatches comparatively to the consensus sequence (-15 to +6)

* http://www.ambion.com/techlib/append/promoter_sequences.html
